# Inspired or entitled? The contingent effects of coworker AI-augmented performance through the lens of social comparison

**DOI:** 10.3389/fpsyg.2026.1969612

**Published:** 2026-10-30

**Authors:** Yi Wang

**Affiliations:** School of Urban Governance and Public Affairs, Suzhou City University, Suzhou, China

**Keywords:** AI-augmented performance, coworker reaction, interpersonal knowledge seeking, psychological entitlement, social comparison theory, task interdependence

## Abstract

**Introduction:**

Research on the interpersonal consequences of workplace AI has mainly examined how observers evaluate the act of AI use. As AI use becomes ordinary, however, use itself loses diagnostic value, and the observable performance produced by AI-assisted work becomes more consequential. Drawing on social comparison theory, we propose that coworker AI-augmented performance is a comparison-relevant cue that yields contingent interpersonal consequences for observers, depending on task interdependence.

**Methods:**

We tested this dual-pathway model in an online between-subjects experiment with full-time employees (*N* = 240) and a three-wave field study of employee–supervisor dyads (*N* = 360).

**Results:**

When task interdependence was high, coworker AI-augmented performance was positively associated with interpersonal knowledge seeking, which in turn increased employee work performance. When task interdependence was low, coworker AI-augmented performance was positively associated with psychological entitlement, which in turn decreased employee work performance. Findings were consistent across the experiment and the field study.

**Discussion:**

Coworker AI-augmented performance can inspire knowledge seeking or invite entitlement, depending on task interdependence. These results highlight both the benefits and perils of AI-augmented performance cues and the need to examine relational work structure in coworker reactions to AI.

## Introduction

AI tools are no longer confined to specialized technical roles. They are entering everyday knowledge work, where employees draft, analyze, and deliver outputs with machine assistance in full view of their colleagues. As this happens, a distinctly interpersonal question comes into focus: how do employees respond when they see coworkers succeed, or struggle, with AI? Existing research has approached this question largely as a problem of social evaluation. Visible AI use invites attributions of reduced effort or diluted competence, and these attributions carry interpersonal costs for users, including less favorable evaluations and less help from coworkers (Reif et al., 2025; Zhou et al., 2025). From that vantage point, AI presents organizations with a familiar trade-off. It can raise task output while lowering the user’s social standing.

Useful as that conversation has been, it is becoming an incomplete guide to the phenomenon it seeks to explain. Even within evaluation research, reactions to AI use are already contingent rather than categorical (Reif et al., 2025). Recent work further shows that peer generative AI adoption can activate constructive and defensive expectancies in parallel (Yu et al., 2026), and that AI use can, under some conditions, reduce the interpersonal burden employees place on teammates and support status conferral (He et al., 2026). The deeper limitation, however, is not simply that positive interpersonal correlates have been underemphasized. It is that this literature still centers a stimulus that is losing diagnostic value. As AI use becomes organizationally ordinary, “whether a coworker uses AI” tells observers less than it once did. What remains visible, and what is more likely to invite social comparison, is the performance that AI-assisted work produces. In this sense, AI-augmented performance invites different judgments regarding how AI can be used in future daily work.

We therefore shift the focal stimulus from AI use to coworker AI-augmented performance: the observable work outcomes a colleague achieves through AI-assisted work. To explore how and when such performance constitutes a comparison-relevant cue, we draw on social comparison theory (Festinger, 1954; Wood, 1989) to propose that a coworker’s visible AI-assisted outcomes provide a concrete reference point for social comparison as people evaluate their own abilities and relative standing against others’ observable accomplishments. SCT holds that upon observing a superior-performing other, individuals engage in upward social comparison, which can produce either an inspirational response that motivates emulation and self-improvement or a self-protective response driven by relative self-evaluation threat (Buunk and Gibbons, 2007; Mussweiler, 2003). We thus propose that coworker AI-augmented performance induces knowledge seeking and psychological entitlement.

A central insight of this theory, however, is that observers do not respond to the same comparison cue in a uniform way. How they respond depends on the relational context linking the observer to the comparison target, and in particular on whether the target’s outcomes are self-relevant for the observer’s own standing and task success (Mussweiler, 2003; Tesser, 1988). In organizational settings, task interdependence is a key structural feature that shapes this self-relevance: it determines whether a coworker’s AI-augmented performance bears on the observer’s own work. When task interdependence is high, the coworker’s performance outcomes are directly consequential to the observer’s own task success; the high-performing AI user is encountered not as a competitor but as a collaborative resource, making knowledge seeking the dominant response. When task interdependence is low, the coworker’s superior performance constitutes a pure relative status threat with no corresponding benefit to the observer’s outcomes, activating psychological entitlement instead. Task interdependence is thus not merely a contextual background variable but the theoretical pivot that determines whether AI-augmented performance is socially generative or socially corrosive.

Building on this framework, we propose that coworker AI-augmented performance is related to observers’ work performance through two opposing mediating pathways contingent upon task interdependence. Under high interdependence, observers are more likely to view the coworker’s AI-assisted success as relevant and attainable, a stance associated with greater interpersonal knowledge seeking—proactively acquiring the methods, practices, and strategies underlying that success—and, in turn, with higher work performance. Under low interdependence, observers are more likely to experience the coworker’s AI-assisted gains as less shared and more undeserved relative to themselves, a stance associated with greater psychological entitlement—a momentary perception that one is deserving of equivalent rewards without equivalent effort (Campbell et al., 2004; Vincent and Kouchaki, 2016)—and, in turn, with lower work motivation and performance. Taken together, we conceptualize coworker AI-augmented performance as a double-edged sword: capable of cultivating a learning workforce or breeding a sense of unearned privilege, depending on the structural context in which it is observed (see Figure 1).

**Figure 1:**
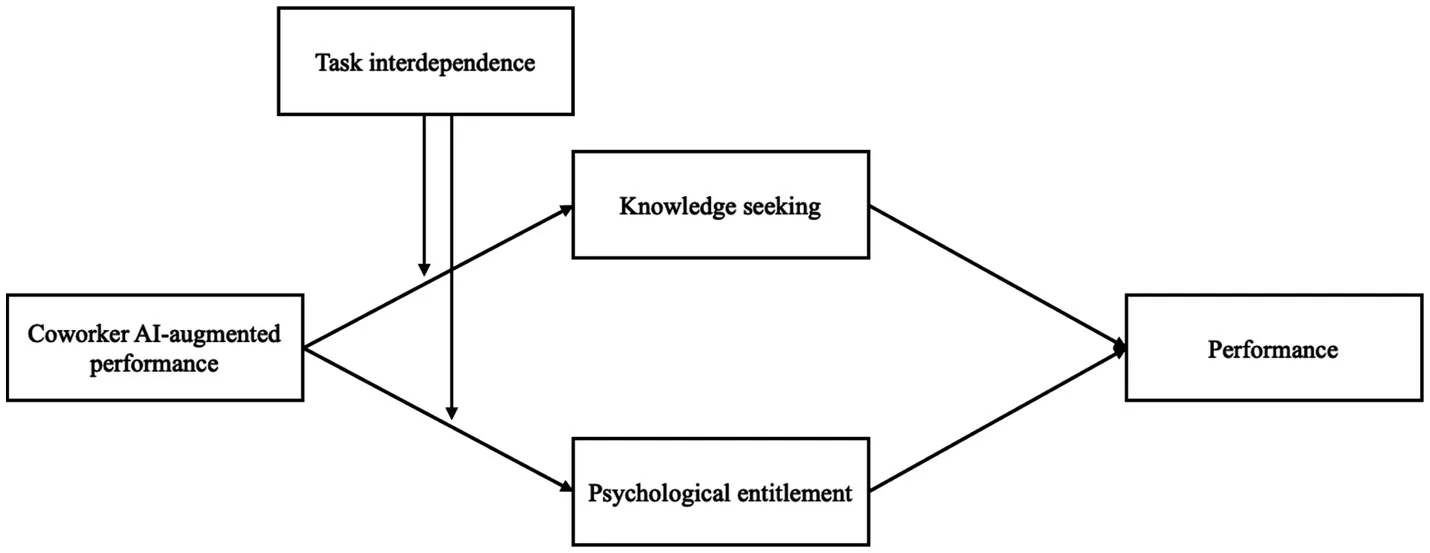
Theoretical model of the current research.

This research makes three theoretical contributions. First, we relocate the focal stimulus in research on coworker reactions to AI from AI use or adoption to coworker AI-augmented performance. Prior work has mainly examined how observers evaluate colleagues who use AI, documenting social costs while beginning to identify contingencies around use and peer adoption (He et al., 2026; Reif et al., 2025; Yu et al., 2026; Zhou et al., 2025). As AI use becomes ordinary, however, use itself loses diagnostic value. Focusing on the observable performance of AI-assisted work extends this literature to a comparison-relevant cue that is likely to matter more as AI diffusion advances. Second, we identify task interdependence as a work-design boundary condition. Social comparison theory suggests that responses to the same comparison cue vary with relational context and self-relevance (Mussweiler, 2003; Tesser, 1988). Task interdependence captures this structural self-relevance at work and helps explain when coworker AI-augmented performance is more likely to be associated with learning-oriented versus self-protective responses. Third, we examine two specific interpersonal pathways: knowledge seeking and psychological entitlement. Moving beyond evaluative judgments of AI users, we show how coworker AI-augmented performance is related to observers’ own work performance through these responses.

## Theory and hypotheses

### Social comparison theory

Social comparison theory (SCT), originally proposed by Festinger (1954), holds that individuals possess a fundamental drive to evaluate their own opinions and abilities by comparing themselves with others. Of particular relevance to the present research is SCT’s recognition that social comparison can elicit two fundamentally opposing responses (Buunk and Gibbons, 2007; Mussweiler, 2003). When a comparison target’s performance is experienced as psychologically proximal and self-relevant, observers may be inspired to emulate the target and close the performance gap—an approach-oriented motivational state associated with learning and behavioral change. When the same performance is instead experienced as a threat to self-evaluation, observers may enter a defensive motivational state characterized by self-protective cognition and disengagement. Critically, SCT does not predict a uniform response to social comparison. Rather, which of these responses is more likely depends on the relational context between observer and target (Mussweiler, 2003; Tesser, 1988). Tesser’s (1988) self-evaluation maintenance model specifies that when an interdependent relationship aligns the interests of observer and target, a high-performing other can reflect positively on the self and encourage emulation; when the relationship is characterized by independence or competition, the same performance can constitute a relative threat to self-standing and encourage defensive self-protection instead.

We propose that SCT offers a theoretically appropriate and parsimonious framework for understanding how coworkers respond to a colleague’s AI-augmented performance. Coworker AI-augmented performance—the level of work outcomes a colleague achieves through AI-assisted work, whether impressive or substandard—creates an observable performance reference point that invites social comparison among observers (Festinger, 1954). Experimental social comparison research commonly induces comparison by presenting high versus low—or outstanding versus less outstanding—comparison standards (Lockwood and Kunda, 1997; Mussweiler et al., 2004; see also Mussweiler, 2003). Following this logic, we treat variation in coworker AI-augmented performance as variation in the strength of the comparative stimulus: higher levels supply a stronger high-performing referent, whereas lower levels supply a weaker one.

Crucially, the same observable performance level is not expected to elicit uniform behavioral reactions; rather, SCT suggests that observers’ responses will diverge depending on the relational context in which comparison takes place. This is precisely the explanatory challenge at the heart of our research: why coworker AI-augmented performance is related to knowledge seeking among some observers yet to psychological entitlement among others. SCT addresses this by specifying that task interdependence—the structural feature that determines whether a coworker’s performance outcomes bear directly on one’s own task success—shapes which comparison response is more likely. Under high interdependence, the coworker functions as a relevant, cooperative reference point, a context associated with approach-oriented responses such as interpersonal knowledge seeking; under low interdependence, the same coworker becomes a competitive comparison standard, a context associated with defensive cognitions such as psychological entitlement. SCT thus helps account for the two opposing mediating pathways in our model and theoretically grounds task interdependence as the boundary condition that channels comparison toward one path or the other.

### The interactive effect of coworker AI-augmented performance and task interdependence on knowledge seeking

When a coworker’s use of AI tools produces observable performance outcomes, those outcomes create an immediate and unavoidable performance reference point for colleagues who witness them. Drawing on social comparison theory (Festinger, 1954), we argue that such observable performance naturally invites comparison: observers assess what the coworker has achieved relative to their own performance, and this comparative assessment sets the stage for a behavioral response. The critical theoretical question, however, is not whether comparison occurs, but which type of comparison process it activates—and it is here that the direction of observers’ behavioral responses, including their motivation to seek knowledge from the coworker, is determined.

SCT suggests that upward performance comparison can inspire approach-oriented behavior toward the comparison target (Buunk and Gibbons, 2007; Collins, 1996). When observers experience a coworker’s performance as informative and motivating rather than threatening, the performance gap is more likely to be interpreted as evidence of an effective and potentially learnable strategy, rather than as a verdict on the observer’s own inadequacy (Mussweiler, 2003). This orientation is associated with interpersonal knowledge seeking—the proactive effort to observe, inquire about, and acquire the methods underlying the coworker’s effective performance (Wilkesmann et al., 2009). When a coworker’s AI-assisted work is visibly effective, observers may therefore come to view that coworker’s approach as a resource to be acquired: they seek out the coworker, ask how they have leveraged AI tools, and invest effort in learning the strategies behind the observed performance (Wang and Noe, 2010). Critically, what invites this response is not the act of AI use per se, but the observable quality of the outcomes it produces—the coworker serves as a performance model whose methods are worth acquiring.

Whether this approach-oriented response is more likely to unfold, however, depends on the relational context in which the comparison takes place. We propose that task interdependence is the structural feature that shapes whether comparison with a high-performing coworker is associated with knowledge seeking. Building on Tesser’s (1988) self-evaluation maintenance model, we argue that when task interdependence is high, the coworker’s performance outcomes directly bear on the observer’s own task success: the two employees share workflow inputs and outputs, meaning that the coworker’s AI-enabled performance quality has tangible consequences for the observer’s own ability to complete their work. Under these conditions, the coworker is experienced not as a competitive reference point but as a functionally aligned collaborator whose success is instrumentally connected to one’s own—a relational framing under which approaching and learning from the coworker becomes psychologically natural (Tesser, 1988). Furthermore, high interdependence substantially increases the instrumental value of knowledge seeking: when a coworker’s effective AI use directly affects shared workflow outcomes, acquiring their methods carries clear and immediate benefits for one’s own performance (Thompson, 1967; Wageman, 1995). The combination of an approach-oriented comparison response and high instrumental incentive is therefore expected to yield a positive relationship between coworker AI-augmented performance and interpersonal knowledge seeking. When task interdependence is low, however, this logic does not hold. The observer’s task outcomes are structurally decoupled from the coworker’s: whatever the coworker achieves with AI assistance has little bearing on the observer’s own deliverables. Under these conditions, the instrumental motivation to seek knowledge from the coworker diminishes substantially, as the practical benefits of doing so are unclear (Wang and Noe, 2010). Moreover, SCT suggests that in the absence of shared interests, comparison is more susceptible to a defensive response—where the coworker’s performance is experienced as a competitive rather than collaborative reference point (Mussweiler, 2003; Tesser, 1988). This defensive orientation is associated with reduced approach behavior toward the comparison target, making interpersonal knowledge seeking less likely. Accordingly, we predict that the positive relationship between coworker AI-augmented performance and knowledge seeking is attenuated when task interdependence is low.

*Hypothesis 1*: Coworker AI-augmented performance and task interdependence will interact to influence interpersonal knowledge seeking such that the relationship will be positive when task interdependence is high and will be weaker when task interdependence is low.

### Knowledge seeking and performance

Having established that coworker AI-augmented performance promotes interpersonal knowledge seeking under conditions of high task interdependence, we next consider how knowledge seeking, once activated, shapes the observer’s own work performance. Interpersonal knowledge seeking reflects a deliberate and effortful investment in acquiring task-relevant information, strategies, and methods from a high-performing colleague (Wilkesmann et al., 2009). When employees actively seek out the working methods that underlie a coworker’s AI-assisted performance—observing how they prompt, verify, and integrate AI outputs into their workflow—they gain access to practical knowledge that is directly applicable to their own tasks. This acquired knowledge reduces uncertainty about effective task execution, expands the observer’s behavioral repertoire, and equips them to produce higher-quality outputs (Argote and Ingram, 2000; Borgatti and Cross, 2003). The relationship between interpersonal knowledge seeking and work performance is thus grounded in a straightforward knowledge acquisition logic: employees who invest effort in learning from effective colleagues accumulate performance-relevant resources that translate into improved task outcomes (Cross and Cummings, 2004; Wang and Noe, 2010).

Connecting this to our moderated framework, the performance benefits of knowledge seeking are contingent upon whether that seeking is more likely to occur in the first place. Coworker AI-augmented performance is expected to be positively related to interpersonal knowledge seeking primarily when task interdependence is high, because shared workflow dependencies both encourage an approach-oriented comparison response and create clear instrumental incentives for knowledge acquisition. When task interdependence is low, the same coworker performance is less likely to motivate knowledge seeking, leaving the potential performance benefits of observational learning unrealized. It follows that the indirect relationship between coworker AI-augmented performance and employee work performance via interpersonal knowledge seeking will be meaningful and positive under high interdependence, where approach-oriented comparison and instrumental learning incentives are both present, but will be attenuated under low interdependence, where neither condition is met. In this way, task interdependence not only shapes the initial comparison response, but also helps determine whether the downstream benefits of social learning ultimately reach the observer’s own performance.

*Hypothesis 2*: *Interpersonal knowledge seeking will mediate the interactive effect of coworker AI-augmented performance and task interdependence on employee work performance such that the indirect effect will be positive when task interdependence is high and will be weaker when task interdependence is low*.

### The interactive effect of coworker AI-augmented performance and task interdependence on psychological entitlement

Coworker AI-augmented performance does not operate in a social vacuum. As established in our theoretical framework, observable performance outcomes produced through AI assistance invite social comparison among colleagues (Festinger, 1954; Wood, 1989). While the preceding section argued that approach-oriented comparison underpins the pathway to knowledge seeking, SCT equally suggests that the same comparative stimulus can elicit a qualitatively different and psychologically more defensive response (Buunk and Gibbons, 2007; Mussweiler, 2003). Under this defensive orientation, the coworker’s performance is experienced not as an informative model to emulate but as a competitive reference point that accentuates the gap between the coworker’s outcomes and one’s own, thereby threatening the observer’s self-evaluation (Tesser, 1988). Such a gap may be experienced as relative deprivation—the sense of being unfairly disadvantaged in standing or deserved outcomes relative to a relevant other (Crosby, 1976). The behavioral and cognitive consequences of this defensive response are therefore fundamentally different from those of an approach-oriented response, and it is through this route that coworker AI-augmented performance becomes related to psychological entitlement.

Psychological entitlement refers to a pervasive sense that one deserves more and is entitled to more than others (Campbell et al., 2004). In the present context, we focus on its state-level manifestation: a momentary perception, invited by situational comparison cues, that one deserves equivalent or superior recognition and rewards relative to a high-performing colleague, regardless of whether one has produced comparable outcomes (Harvey and Martinko, 2009; Vincent and Kouchaki, 2016). This state of entitlement can function as a cognitive response to the relative deprivation created by defensive upward comparison. When observers cannot match the performance level of an AI-augmented coworker, and when the relational context does not provide an alternative means of incorporating that performance into a positive self-evaluation, entitlement cognitions may help restore a sense of equity: rather than improving performance or acquiring the coworker’s methods, the observer instead reasons that they deserve comparable rewards by virtue of their general worth and contributions—effectively closing the psychological gap without engaging in behavioral change (Campbell et al., 2004; Harvey and Martinko, 2009).

We propose that task interdependence is the structural condition that shapes whether psychological entitlement is more likely in response to coworker AI-augmented performance. When task interdependence is low, observers and the AI-augmented coworker occupy structurally independent positions: their outcomes are decoupled, their workflows do not intersect, and the coworker’s performance gains carry no direct benefit for the observer’s own tasks. Under these conditions, the coworker’s performance functions as a pure relative comparison standard—a benchmark against which the observer’s own standing is assessed and found wanting, making relative deprivation more salient. With no shared interest to reframe the coworker as an ally, and no instrumental pathway through which the coworker’s success might also become the observer’s success, a defensive comparison response is more likely (Tesser, 1988). The resulting self-evaluation threat is associated with entitlement cognitions: the observer asserts a sense of deserving that compensates psychologically for the performance gap, without requiring the observer to close that gap behaviorally (Vincent and Kouchaki, 2016). Accordingly, we expect that coworker AI-augmented performance will be positively related to observer psychological entitlement when task interdependence is low.

When task interdependence is high, however, this dynamic is substantially attenuated. Under conditions of shared workflow dependency, the coworker’s AI-assisted performance outcomes are instrumentally connected to the observer’s own task success. Drawing on Tesser’s (1988) self-evaluation maintenance model, we argue that this shared interest reframes the comparison: the coworker’s success is no longer a zero-sum signal of relative disadvantage but is partially incorporated into the observer’s self-concept through their collaborative relationship. The self-evaluation threat that would otherwise invite relative deprivation—and subsequently entitlement—is buffered by the perceived alignment of interests, shifting comparison toward approach-oriented responses and reducing the motivational pressure for entitlement-related reasoning. Consequently, the positive relationship between coworker AI-augmented performance and psychological entitlement is expected to be weaker under high task interdependence.

*Hypothesis 3*: Coworker AI-augmented performance and task interdependence will interact to influence employee psychological entitlement such that the relationship will be positive when task interdependence is low and will be weaker when task interdependence is high.

### Psychological entitlement and performance

Psychological entitlement impairs work performance through a motivational mechanism rooted in the decoupling of effort from expected reward. Entitlement reflects a belief that one deserves recognition and outcomes by virtue of inherent worth, independent of the actual effort or quality of work one produces (Campbell et al., 2004). When this belief is activated, the instrumental logic that typically connects effort investment to performance outcomes is disrupted: if rewards are perceived as owed regardless of contribution, the motivation to invest discretionary effort into one’s work is correspondingly diminished (Harvey and Martinko, 2009; Naumann et al., 2002). Entitled employees are thus less likely to engage in the sustained effort, attention, and task persistence that high performance requires, and more likely to conserve resources in anticipation of rewards they believe will arrive regardless. The result is a systematic reduction in observable work performance—not because the individual lacks ability, but because entitlement cognitions have eroded the motivational foundation on which effortful performance depends (Harvey et al., 2014).

The performance-impairing consequences of psychological entitlement are more likely specifically when a defensive comparison response is invited—that is, when task interdependence is low and coworker AI-augmented performance constitutes a self-evaluative threat rather than a shared resource. Under low interdependence, coworker AI-augmented performance is positively related to psychological entitlement, which in turn is negatively related to the observer’s own work performance. This full indirect pathway represents the defensive chain through which the same observable performance stimulus that is associated with knowledge seeking under high interdependence is instead associated with motivational withdrawal under low interdependence. Conversely, when task interdependence is high, defensive comparison responses are attenuated, entitlement is less likely, and the performance-impairing indirect pathway is correspondingly weaker. The moderated mediation through psychological entitlement thus constitutes the dark counterpart to the learning pathway described in Hypothesis 2: it captures the conditions under which observing a coworker’s AI-assisted performance is related to depleted rather than developed observer performance capacity.

*Hypothesis 4*: Psychological entitlement will mediate the interactive effect of coworker AI-augmented performance and task interdependence on employee work performance such that the indirect effect will be negative when task interdependence is low and will be weaker when task interdependence is high.

## Overview of the current research

To test our theoretical model, we conducted two studies, including an experimental study (i.e., Study 1) and a multiwave field study (i.e., Study 2). In Study 1, we experimentally examined the interactive effect of coworker AI-augmented performance and task interdependence on employee knowledge seeking (i.e., Hypothesis 1) and psychological entitlement (i.e., Hypothesis 3), our two key hypotheses. In Study 2, we used a multiwave design to test our full model (Hypotheses 1–4) in a field setting to maximize external validity. This multimethod design (i.e., experimental and field studies) helps establish the internal and external validity of our findings (Ju et al., 2019; Qin et al., 2015). Data were analyzed using SPSS (Version 25) and Mplus (Version 8.4).

## Study 1 method

### Participants and procedure

We conducted a study with full-time employees drawn from a subject pool comprising various companies in the technology industry in China. Participants were presented with an invitation letter that included information about the study (e.g., duration, procedure, and compensation). Participation was voluntary, and respondents were assured that their personal information would be kept confidential. The sample size for this study was determined through statistical power analysis using G*Power software (Faul et al., 2009). Assuming a medium between-conditions effect size of d = 0.50, 210 participants were required to achieve an *α* level of 0.05 and statistical power of 1–*β* = 0.80. To account for potential missing data, we predetermined a recruitment target of 60 participants per condition.

Therefore, we invited 240 full-time employees to participate in a between-subjects experiment (coworker AI-augmented performance: high vs. low; task interdependence: high vs. low) via the Credamo online platform. We randomly assigned participants to one of the four experimental conditions. All participants passed the attention check, resulting in a final sample of 240 participants (141 male participants; average age = 37.98, SD = 9.94).

### Experimental design

#### Coworker AI-augmented performance manipulation

Coworker AI-augmented performance was manipulated as a between-subjects factor with two levels (high vs. low). All participants read a common scenario in which they were asked to imagine themselves as an employee on a business team at a mid-sized company where generative AI tools had become available for daily work. Participants were further told that one of their coworkers—referred to as Xiao Li—held a similar position and had recently used AI tools to complete a work assignment. Importantly, all conditions held constant that Xiao Li used AI and carefully reviewed, verified, and revised the AI output before submission; the manipulation varied only the observable quality of Xiao Li’s final AI-assisted work outcomes.

In the high coworker AI-augmented performance condition, participants read that, after this careful review process, Xiao Li’s final deliverable was accurate, complete, and met the task quality standard (e.g., a very low error rate and successful quality check). In the low coworker AI-augmented performance condition, participants read that Xiao Li followed the same careful review process, but the final deliverable still contained multiple inaccuracies or incompletenesses and did not meet the quality standard (e.g., a higher error rate and failure to pass the quality check). Thus, the conditions differed in performance outcomes. To check whether the performance conditions also differed in the attributes confounded in the original design, participants rated Xiao Li’s effort (“This coworker invested considerable effort in completing the work”), careful review of AI output (“When using AI, this coworker carefully reviewed and checked the AI output”), and responsible AI use (“This coworker used AI tools responsibly”).

##### Task interdependence manipulation

Task interdependence was manipulated as a structural feature of the work design. In the high task interdependence condition, participants read that their work and Xiao Li’s work were sequentially linked in the same workflow: Xiao Li completed an upstream module whose output served as a necessary input for the participant’s downstream tasks, such that completing a full deliverable required integrating Xiao Li’s output. In the low task interdependence condition, participants read that they and Xiao Li worked on parallel, separate task modules and that completing one’s own work did not require Xiao Li’s output as a necessary input. Critically, the interdependence scenarios described structural dependence only.

#### Measures

All measures were translated from English into Chinese using the back-translation procedure recommended by Brislin (1986). Participants rated each item on a 7-point Likert scale (1 = strongly disagree, 7 = strongly agree).

##### Knowledge seeking

We adopted Wilkesmann et al. (2009)’s four-item scale to measure the focal employees’ knowledge seeking from the coworker. A sample item is “I turned to Xiao Li for advice regarding special procedures for efficiently completing work with the help of AI so that I learn them”. In this study, Cronbach’s *α* was 0.88.

##### Psychological entitlement

We adopted Campbell et al. (2004)’s nine-item scale to measure the focal employees’ psychological entitlement. A sample item is “I honestly feel I’m just more deserving than Xiao Li”. In this study, Cronbach’s *α* was 0.91.

## Study 1 results

### Manipulation check

We used two items adapted from Oldham and Cummings (1996) to measure coworker AI-augmented performance. The items are “With the help of AI tools, Xiao Li meets performance expectations”. As expected, participants in the experimental condition reported significantly greater coworker AI-augmented performance (*M* = 5.34, SD = 0.57) than those in the control condition (*M* = 2.81, SD = 0.62), *t*(236) = 33.05, *p* < 0.001.

We used two items adapted from Van der Vegt and Janssen (2003) to measure task interdependence. The items are “Based on the scenario, I need to collaborate with Xiao Li to perform my job well”. As expected, participants in the experimental condition reported significantly greater task interdependence (*M* = 5.46, SD = 0.56) than those in the control condition (*M* = 2.88, SD = 0.54), *t*(238) = 36.23, *p* < 0.001.

### Balance check

These ratings did not differ between the high- and low-performance conditions. For effort, the means were *M* = 5.10 (SD = 0.70) and *M* = 5.17 (SD = 0.67), *t*(237) = 0.75, *p* = 0.451. For careful review, the means were *M* = 5.10 (SD = 0.64) and *M* = 5.20 (SD = 0.66), *t*(238) = 1.20, *p* = 0.233. For responsible AI use, the means were *M* = 5.17 (SD = 0.65) and *M* = 5.13 (SD = 0.69), *t*(237) = 0.39, *p* = 0.700.

### Hypothesis testing

The interactive effect of coworker AI-augmented performance and task interdependence on knowledge seeking was significant (*b* = 1.39, *p* < 0.001). Simple slope tests further indicated that this relationship was significant and positive when task interdependence is high (*b* = 1.63, *t* = 13.14, *p* < 0.001), and was weaker when task interdependence is low (*b* = 0.25, *t* = 1.98, *p* = 0.049; see Figure 2). Under high interdependence, knowledge seeking was higher in the high-performance condition (*M* = 5.23, SD = 0.64) than in the low-performance condition (*M* = 3.60, SD = 0.66), *t*(118) = 13.74, *p* < 0.001. Under low interdependence, the corresponding means were *M* = 3.41 (SD = 0.66) and *M* = 3.17 (SD = 0.76), *t*(116) = 1.90, *p* = 0.060. These findings provide support for Hypothesis 1.

**Figure 2:**
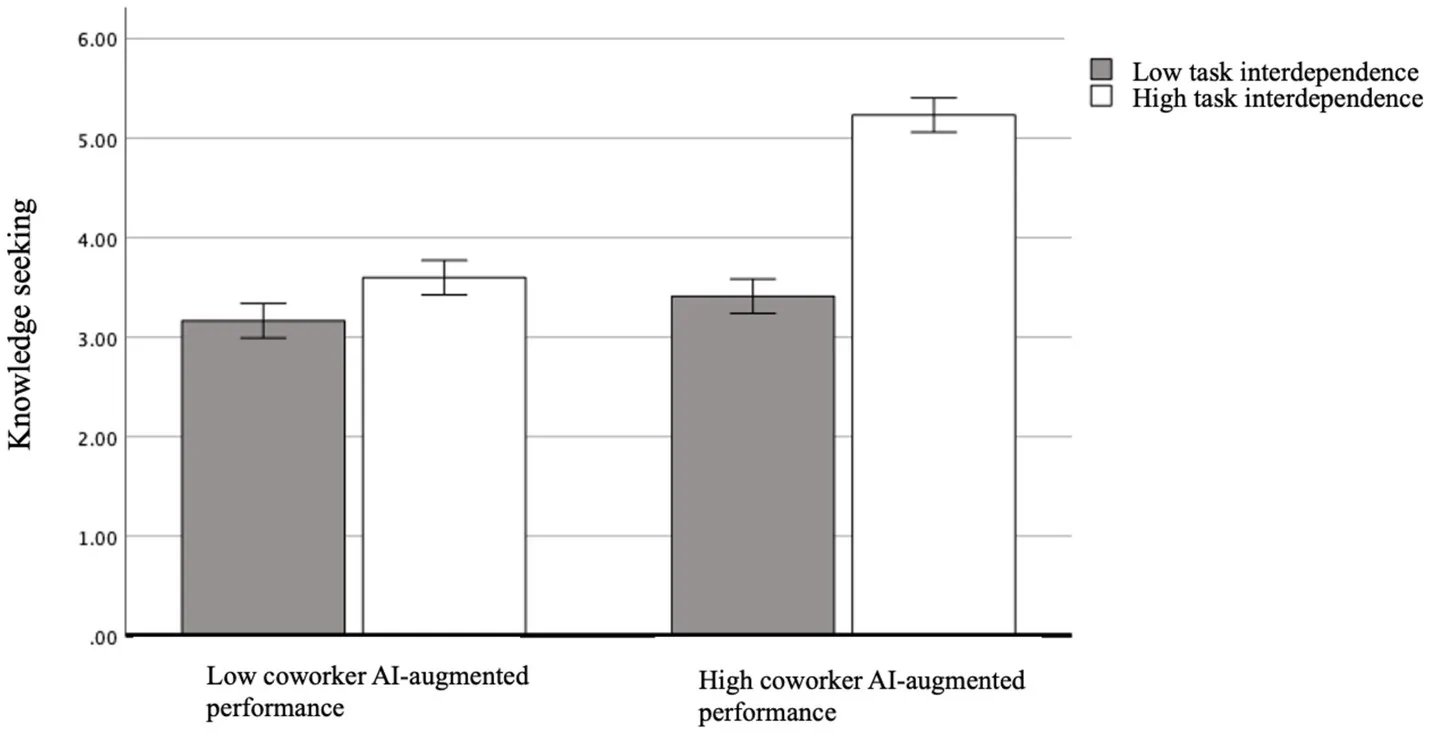
The interactive effect of coworker AI-augmented performance and task interdependence on knowledge seeking in Study 1.

The interactive effect of coworker AI-augmented performance and task interdependence on psychological entitlement was significant (*b* = −1.21, *p* < 0.001). Simple slope tests further indicated that this relationship was significant and positive when task interdependence is low (*b* = 1.39, *t* = 13.25, *p* < 0.001), but was not significant when task interdependence was high (*b* = 0.18, *t* = 1.72, *p* = 0.087; see Figure 3). Under low interdependence, psychological entitlement was higher in the high-performance condition (*M* = 4.52, SD = 0.55) than in the low-performance condition (*M* = 3.14, SD = 0.58), *t*(118) = 13.50, *p* < 0.001. Under high interdependence, the corresponding means were *M* = 3.33 (SD = 0.57) and *M* = 3.15 (SD = 0.60), *t*(118) = 1.69, *p* = 0.094. These findings provide support for Hypothesis 3.

**Figure 3:**
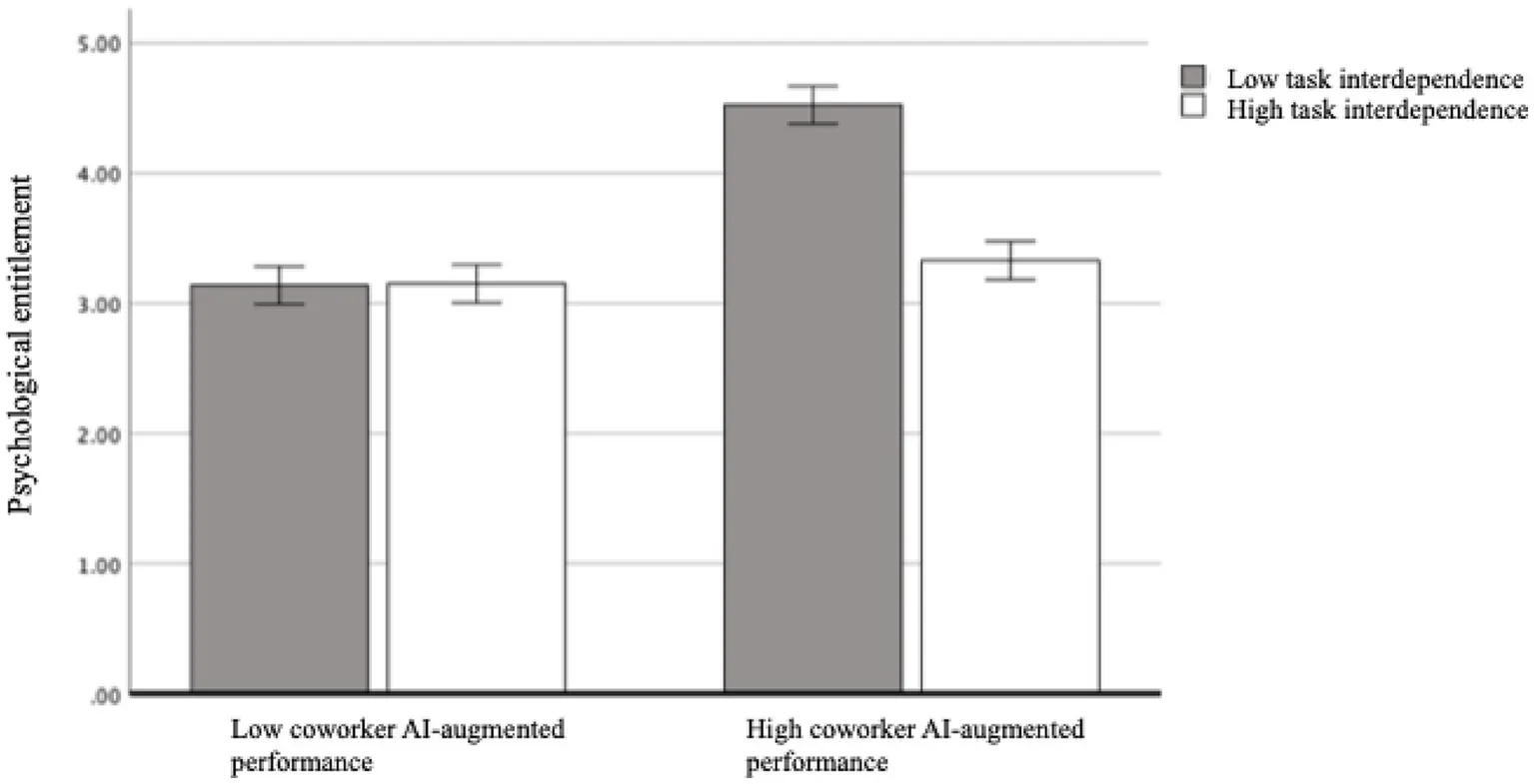
The interactive effect of coworker AI-augmented performance and task interdependence on psychological entitlement in Study 1.

## Study 2 method

### Participants and procedure

The Study 2 sample consisted of independently recruited employee–supervisor dyads who worked together regularly in their organizations. Focal employees were not nested within larger teams in our sample, and each supervisor was paired with only one subordinate. Accordingly, supervisor ratings were not clustered within supervisors or teams, the observations are independent at the dyad level. At Time 1, each employee also identified one particular coworker with whom they worked regularly and completed the coworker-referenced measures (coworker AI-augmented performance and task interdependence) with respect to that person. Employees selected this coworker themselves; we did not assign coworkers to respondents. The same focal coworker was not selected by more than one employee. Employee responses were therefore not nested within coworkers. The first-round survey was conducted in November 2025, the second-round survey was conducted in December 2025, and the third-round survey was conducted in January 2026. Before we distributed the first-round questionnaire, we conveyed high confidentiality, voluntary participation, and that the surveys would only be used for academic research. Participants received ¥5 for three round surveys. Our initial sample was 380 employee and supervisor dyads. At Time 1, participants completed questionnaires regarding demographics, perceived coworker AI-augmented performance and task interdependence with the focal coworker in the last month. At Time 2, participants completed questionnaires regarding their knowledge seeking and psychological entitlement. At Time 3, supervisors completed questionnaires regarding their subordinates’ performance in the past month. Employee and supervisor responses were matched by unique dyad identifiers across waves. Of the 380 dyads initially recruited, 20 did not complete the full three-wave procedure because participants withdrew from the study (attrition rate = 5.3%). We did not exclude any completed cases for other reasons, and we did not impute missing data; incomplete dyads were simply unavailable for matching. The final matched analytic sample therefore comprised 360 employee–supervisor dyads.

### Measures

All measures were translated into Chinese by the author using the back-translation procedures recommended by Brislin (1986).

#### Coworker AI-augmented performance

We adopted Van Dyne and LePine (1998)’s four-item scale to measure perceived coworker AI-augmented performance. A sample item is “With the help of AI tools, this particular co-worker fulfills the responsibilities specified in his/her job description”. In this study, Cronbach’s *α* was 0.85.

#### Task interdependence

We adopted Van der Vegt and Janssen (2003)’s five-item scale to measure perceived task interdependence. A sample item is “I need to collaborate with this coworker to perform my job well”. In this study, Cronbach’s *α* was 0.87.

#### Knowledge seeking

As in Study 1, we adopted Wilkesmann et al. (2009)’s four-item scale to measure the focal employees’ knowledge seeking from the coworker. A sample item is “I turned to this coworker for advice regarding special procedures for efficiently completing work with the help of AI so that I learn them”. In this study, Cronbach’s *α* was 0.84.

#### Psychological entitlement

As in Study 1, we adopted Campbell et al. (2004)’s nine-item scale to measure the focal employees’ psychological entitlement. Although the original scale was developed as an individual-difference measure, organizational research commonly assesses situationally invited entitlement by retaining the core deservingness content of the PES while situating responses in a specific interpersonal context (e.g., Vincent and Kouchaki, 2016; see also Zitek and Vincent, 2015). Consistent with this mainstream approach to state operationalization, we (a) provided a situational stem asking participants to report their feelings at the present moment with reference to the focal coworker’s AI-augmented performance, and (b) adapted item referents from a general “others” framing to the specific coworker. A sample item is “I honestly feel I’m just more deserving than this coworker”. In this study, Cronbach’s *α* was 0.91.

#### Performance

We adopted Oldham and Cummings (1996)’s three-item scale to measure individual employees’ performance. A sample item is “Please rate the QUALITY of work done by employee”. In this study, Cronbach’s *α* was 0.76.

#### Control variables

In testing the proposed model, we controlled for participants’ age, gender, educational level, and organizational tenure.

### Analytic strategy

We used ordinary least squares (OLS) to test our hypotheses. To reduce collinearity, all explanatory variables were grand-mean centered (Hofmann and Gavin, 1998). To test the moderated mediation hypotheses, we used Hayes’s (2013) PROCESS macro with bias-corrected bootstrapping based on 20,000 resamples to construct 95% confidence intervals and test the indirect effects at high (1 SD above) and low (1 SD below) levels of the moderator (Edwards and Lambert, 2007; Hayes, 2015).

## Study 2 results

Descriptive statistics and correlations are presented in Table 1. Prior to hypothesis testing, we first conducted a set of CFAs to ensure that our key constructs (i.e., coworker AI-augmented performance, task interdependence, knowledge seeking, psychological entitlement, and performance) had satisfactory discriminant validity. As shown in Table 2, the five-factor model fit the data adequately (*χ*^2^ = 556.86, df = 265, CFI = 0.93, TLI = 0.92, RMSEA = 0.06, SRMR = 0.07) and better than the four-factor model (Δ*χ*^2^ = 575.24, Δdf = 4, *p* < 0.001), the three-factor model (Δ*χ*^2^ = 1125.32, Δdf = 7, *p* < 0.001), the two-factor model (Δ*χ*^2^ = 1729.17, Δdf = 9, *p* < 0.001), and single factor model (Δ*χ*^2^ = 1951.92, Δdf = 10, *p* < 0.001).

**Table 1:** Descriptive statistics and correlations among variables in Study 2.

Variables		Mean	SD	1	2	3	4	5	6	7	8	9
1	Age	38.69	9.97									
2	Gender	0.46	0.50	−0.08								
3	Educational level	2.49	0.71	0.10	−0.02							
4	Organizational tenure	4.39	2.94	0.01	−0.06	0.09						
5	Coworker AI-augmented performance	3.52	0.78	0.08	−0.001	−0.11*	−0.04	(0.85)				
6	Task interdependence	3.48	0.78	−0.04	−0.07	0.00	0.00	0.01	(0.87)			
7	Knowledge seeking	3.51	0.77	0.05	0.01	−0.01	−0.05	0.20**	−0.15**	(0.84)		
8	Psychological entitlement	3.54	0.68	0.00	−0.03	−0.04	0.00	0.10	0.10	−0.18**	(0.91)	
9	Performance	3.52	0.72	−0.01	−0.02	−0.04	0.04	0.09	−0.10	0.32**	−0.28**	(0.76)

*N* = 360. Gender was coded 0 = male and 1 = female. Education was coded as 1 = high school or below, 2 = bachelor’s degree, 3 = master’s degree, and 4 = doctoral degree. Cronbach’s *α* coefficients were reported along the diagonal in bold.

*p < 0.05, *^*^p< 0.01 (two–tailed).

**Table 2:** Comparison of measurements in Study 2.

Model	*χ*^2^(*df*)	CFI	TLI	RMSEA	Δ*χ*^2^ (*df*)	SRMR
Model 1	556.86 (265)	0.93	0.92	0.06		0.07
Model 2	1132.10 (269)	0.78	0.76	0.09	575.24***(4)	0.12
Model 3	1682.18 (272)	0.64	0.61	0.12	1125.32***(7)	0.14
Model 4	2286.03 (274)	0.49	0.44	0.14	1729.17***(9)	0.15
Model 5	2508.78 (275)	0.43	0.38	0.15	1951.92*** (10)	0.16

Model 1, a five-factor model that included coworker AI-augmented performance (CAIP), task interdependence (TI), knowledge seeking (KS), psychological entitlement (PE), and performance (PER). Model 2, a four-factor model (CAIP and TI were combined). Model 3, a three-factor model (CAIP and TI were combined, KS and PE were combined). Model 4 = a two-factor model (CAIP, TI, KS, and PE were combined). Model 5 = the single-factor model (all factors were combined).

Table 3 summarizes the results of path analysis. As shown in Table 3, the interaction effect of coworker AI-augmented performance and task interdependence on knowledge seeking is significant and positive (*β* = 0.42, *p* < 0.001). As depicted in Figure 4, simple slopes tests revealed that the relationship between coworker AI-augmented performance and knowledge seeking was significant and positive when task interdependence is high (*b* = 0.50, *t* = 7.48, *p* < 0.001) but was significantly negative when task interdependence was low (*b* = −0.16, *t* = −2.37, *p* < 0.05). Thus, Hypothesis 1 was supported.

**Table 3:** Path analysis results in Study 2.

Variable	Knowledge seeking		Psychological entitlement		Performance	
	Coeff. (SE)	*p*-value	Coeff. (SE)	*p*-value	Coeff. (SE)	*p*-value
Intercept	3.32 (0.23)	< 0.001	3.73 (0.21)	< 0.001	3.72 (0.35)	< 0.001
Age	0.00 (0.00)	0.67	−0.00 (0.00)	0.99	−0.00 (0.00)	0.67
Gender	0.02 (0.07)	0.77	−0.04 (0.07)	0.54	−0.03 (0.07)	0.62
Educational level	0.05 (0.05)	0.33	−0.05 (0.05)	0.28	−0.05 (0.05)	0.34
Organizational tenure	−0.01 (0.01)	0.42	−0.00 (0.01)	0.99	0.02 (0.01)	0.19
Coworker AI-augmented performance	0.20 (0.05)	< 0.001	0.08 (0.04)	0.07	0.06 (0.05)	0.20
Task interdependence	−0.14 (0.05)	0.003	0.09 (0.04)	0.05		
Coworker AI-augmented performance × Task interdependence	0.42 (0.06)	< 0.001	−0.31 (0.06)	< 0.001		
Knowledge seeking					0.25 (0.05)	< 0.001
Psychological entitlement					−0.26 (0.05)	< 0.001

*N* = 360. Coeff., Unstandardized path coefficients; SE, standard error. The results remain stable when the control variables are excluded from the analyses.

**Figure 4:**
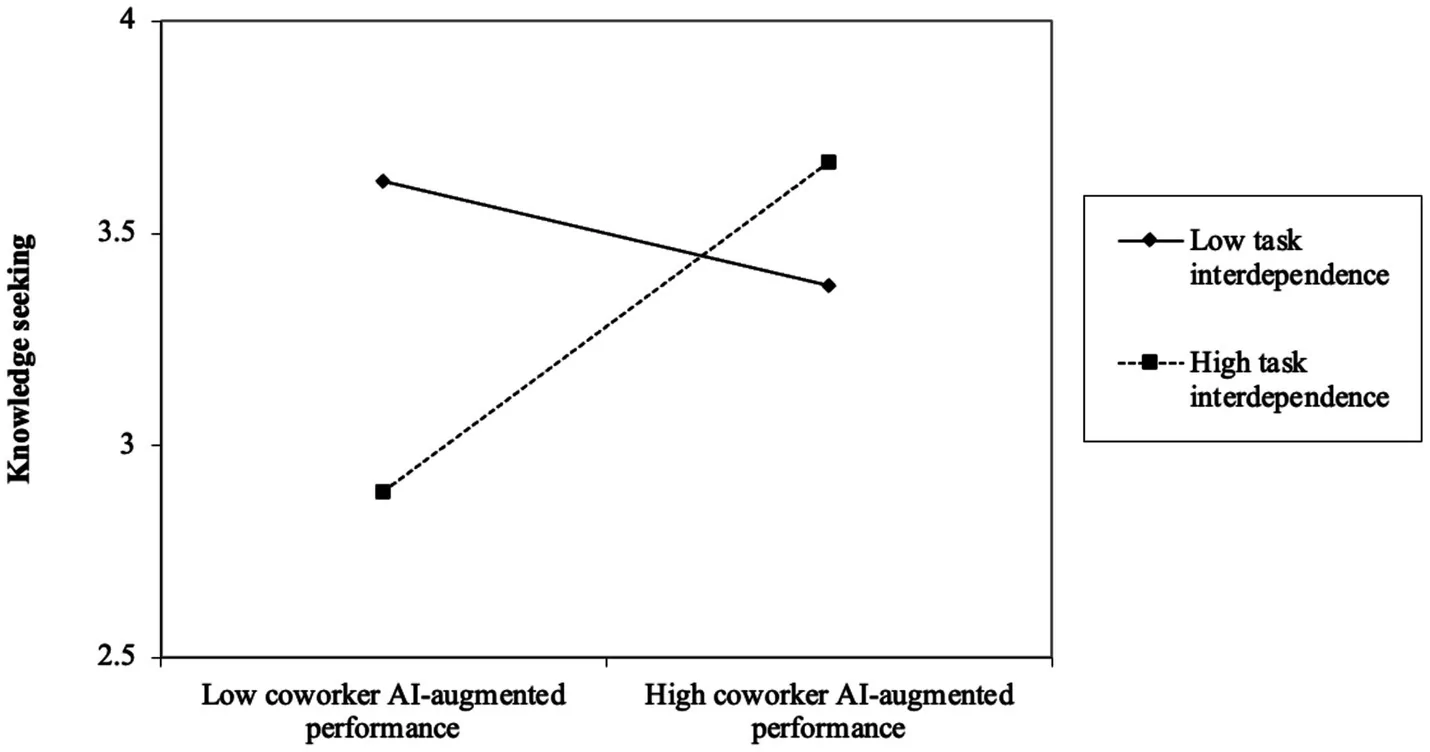
The interactive effect of coworker AI-augmented performance and task interdependence on knowledge seeking in Study 2.

The interaction effect of coworker AI-augmented performance and task interdependence on psychological entitlement is significant and negative (*β* = −0.31, *p* < 0.001), supporting Hypothesis 3. As depicted in Figure 5, simple slopes tests revealed that the relationship between coworker AI-augmented performance and psychological entitlement was significant and positive when task interdependence is low (*b* = 0.33, *t* = 5.32, *p* < 0.001) but was significantly negative when task interdependence was high (*b* = −0.15, *t* = −2.44, *p* < 0.05). Thus, Hypothesis 3 was supported.

**Figure 5:**
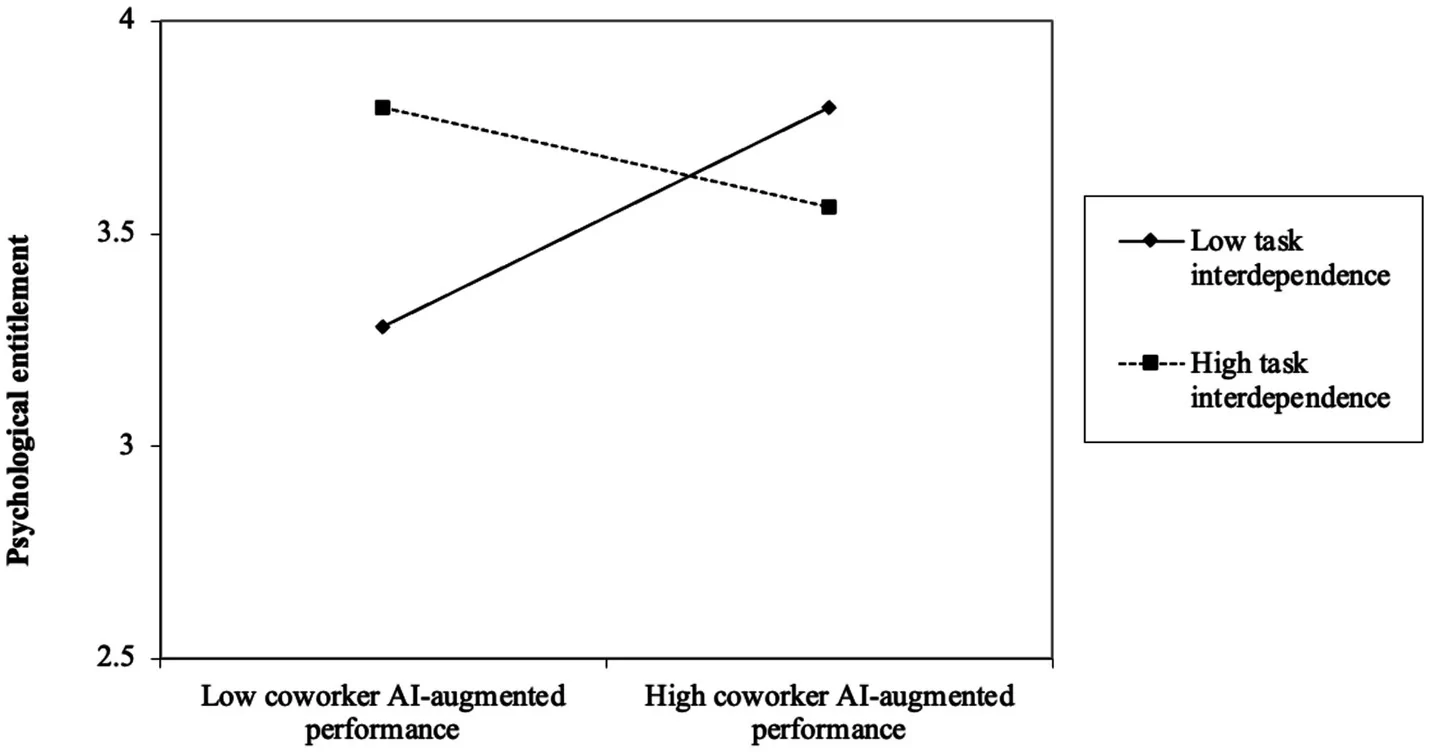
The interactive effect of coworker AI-augmented performance and task interdependence on psychological entitlement in Study 2.

To test the conditional indirect effects, we employed bias-corrected bootstrap procedures with 20,000 resamples. The bootstrap analysis results show that the indirect relationship between coworker AI-augmented performance and performance via knowledge seeking is not significant when task interdependence is low (indirect effect = −0.03, 95%CI = [−0.07, 0.00]) and is positive when task interdependence is high (indirect effect = 0.13, 95%CI = [0.07, 0.20]), supporting Hypothesis 2. The indirect relationship between coworker AI-augmented performance and performance via psychological entitlement is negative when task interdependence is low (indirect effect = −0.08, 95%CI = [−0.13, −0.04]) and is positive when task interdependence is high (indirect effect = 0.04, 95%CI = [0.01, 0.08]), supporting Hypothesis 4.

To formally test moderated mediation, we also estimated the index of moderated mediation for each pathway using bias-corrected bootstrapping with 20,000 resamples. For the knowledge-seeking pathway, the index of moderated mediation was significant (index = 0.11, BootSE = 0.03, 95% CI = [0.06, 0.16]). For the psychological-entitlement pathway, the index of moderated mediation was also significant (index = 0.08, BootSE = 0.02, 95% CI = [0.04, 0.13]). These results indicate that the indirect effects of coworker AI-augmented performance on work performance via knowledge seeking and via psychological entitlement both vary significantly with task interdependence.

## General discussion

Drawing upon social comparison theory, we found that coworker AI-augmented performance is a double-edged sword whose interpersonal consequences are contingent upon task interdependence. When interdependence is high, observers engage in knowledge seeking from AI-augmented colleagues and translate that learning into improved work performance. When interdependence is low, the performance signal fosters psychological entitlement, which erodes observers’ own motivation and undermines their performance. By revealing how coworker AI-augmented performance is associated with opposing interpersonal responses depending on the structural context of work, we hope this study opens new conversations about the organizational conditions under which visible AI-assisted performance is more likely to diffuse learning rather than invite defensive reactions.

### Implications for theory

The present research advances theory on the interpersonal consequences of workplace AI in three ways. First, it relocates the unit of analysis from AI use to AI-augmented performance. Much of the emerging literature has treated the act of using AI, or the intensity of AI collaboration and adoption, as the primary social stimulus, documenting evaluation penalties, reduced helping, and related interpersonal costs (Reif et al., 2025; Wu and Jiao, 2026; Zhou et al., 2025). That work has been important in showing that AI reliance is socially meaningful. Yet as AI tools become standard practice, “who uses AI” becomes a weaker diagnostic cue. Our findings suggest that observers also respond to what AI-assisted work produces. By showing that coworker AI-augmented performance is associated with distinct interpersonal responses depending on task interdependence, we extend the conversation from use-based social evaluation to performance-based social comparison. This reframing does not deny the value of prior work on AI-use penalties or dual adoption signals (He et al., 2026; Yu et al., 2026). Rather, it specifies a complementary stimulus that is likely to gain importance as AI use itself loses distinctiveness.

Second, our results highlight task interdependence as a theoretically grounded work-design contingency. Prior studies have identified boundary conditions such as competitive climate, attribution patterns, and observer characteristics that shape reactions to AI use or peer adoption (Wu and Jiao, 2026; Yu et al., 2026). We add a structural work-design variable that follows directly from social comparison theory’s emphasis on relational context and self-relevance (Mussweiler, 2003; Tesser, 1988). When interdependence is high, a coworker’s AI-augmented performance is more closely tied to the observer’s own task requirements, and the performance cue is positively related to interpersonal knowledge seeking and subsequent performance. When interdependence is low, the same cue is more weakly coupled to shared task demands and is positively related to psychological entitlement and lower subsequent performance. Task interdependence thus does more than amplify an existing effect; it helps explain which interpersonal pathway is more likely to emerge around the same performance signal.

Third, we move from evaluative reactions toward two concrete observer pathways: knowledge seeking and psychological entitlement. Existing research has largely focused on how AI users are judged or treated by others, such as competence, morality, helping, and compensation-related evaluations (Kim et al., 2026; Reif et al., 2025; Zhou et al., 2025). Our model directs attention to what observers do and feel in response to a coworker’s AI-augmented performance, and to how those responses are related to observers’ own work performance. The knowledge-seeking pathway speaks to the potential for social learning around AI-assisted success under interdependent work. The entitlement pathway speaks to a self-protective response that may accompany visible AI-augmented performance when interdependence is low. Together, these pathways offer a more behaviorally specific account of how AI-related performance cues travel through team relationships, without requiring the claim that coworker reactions were previously understood as uniformly negative.

### Implications for practice

The findings offer practical insight for organizations introducing AI into everyday work. Our results suggest that task interdependence is an important contextual condition shaping how employees respond to coworkers’ AI-augmented performance. In more interdependent workflows, visible AI-augmented performance appears more likely to be associated with interpersonal knowledge seeking and subsequent performance gains. In less interdependent settings, the same performance cue appears more likely to be associated with psychological entitlement and weaker performance. Managers may therefore find it useful to consider existing interdependence when anticipating the interpersonal consequences of AI-enabled productivity, rather than treating AI-augmented performance as uniformly beneficial or harmful. While we do not mean that increasing task interdependence is necessarily an effective AI-related work-design intervention. Increasing interdependence can also create substantial coordination costs and is not equally desirable across work systems. A more appropriate implication is diagnostic: organizations should attend to the interdependence structure in which AI-augmented performance becomes visible, monitor whether learning-oriented or self-protective responses are emerging, and tailor support accordingly. Managers should also recognize that interdependence may coexist with disputes over credit and rewards; attending to contribution attribution remains important even when collaborative learning is desirable.

Conversely, our findings highlight a risk that is easily overlooked in organizations where employees work in parallel on largely independent tasks. Under such conditions, visible AI-augmented performance is likely to foster entitlement cognitions that reduce employees’ own work motivation without any corresponding benefit to team coordination. For organizations structured around individual accountability and competitive evaluation, the unmanaged diffusion of AI performance signals may therefore produce unintended motivational costs alongside its productivity gains. Managers in such contexts may consider supplementary interventions—such as structured peer learning sessions, explicit knowledge-sharing norms, or rotating collaboration arrangements—to introduce sufficient relational context to buffer the psychological entitlement effect and redirect comparison energy toward learning.

Finally, these findings caution against uniform AI adoption narratives. The assumption that AI-enhanced performance by any team member will raise the performance floor for all members overlooks the social comparison dynamics documented here. HR practitioners designing AI upskilling programs and performance management systems should account for whether the contexts in which AI tools are deployed are structurally conducive to learning diffusion, and should monitor for signs of entitlement-driven disengagement—particularly in low-interdependence roles—as an unintended consequence of visible AI performance disparities within teams.

### Limitations and future directions

First, our designs constrain the causal claims that can be drawn from the findings. Study 1 experimentally manipulates coworker AI-augmented performance and task interdependence and thus supports causal inference regarding their interactive effects on knowledge seeking and psychological entitlement, whereas Study 2’s multiwave field design strengthens external validity but remains correlational for the full mediated model. Relatedly, because AI use was held constant and only the performance of an AI-using coworker varied, the present evidence speaks to how employees respond to coworker performance within an AI-enabled work context. It does not establish that these social comparison dynamics are caused by AI assistance per se, as opposed to high versus low coworker performance more generally. In addition, we operationalize the comparative stimulus as absolute coworker AI-augmented performance rather than relative standing or perceived comparison direction, so the findings speak to reactions to stronger versus weaker performance standards rather than to verified upward comparison for every observer. Future research could strengthen causal inference by incorporating non-AI comparison conditions, assessing relative performance or perceived comparison direction, and using complementary designs that more fully isolate these interpretive alternatives.

Second, the present model does not account for stable individual characteristics that may condition the strength or direction of social comparison responses. Individual differences such as learning goal orientation, competitive dispositional orientation, prior AI experience, and trait-level psychological entitlement may systematically attenuate or amplify the pathways documented here. For instance, employees high in learning orientation may still engage in knowledge seeking even under low interdependence, whereas those high in trait entitlement may be especially prone to defensive deservingness cognitions. Relatedly, although we adapted Campbell et al.’s (2004) Psychological Entitlement Scale to assess state, coworker-referenced entitlement—consistent with common practice in organizational research—adapted scores may still partly reflect chronic entitlement tendencies alongside momentary deservingness. Because trait and state entitlement share overlapping content, future research could sharpen the model by measuring trait entitlement as a control or moderator, using experience-sampling designs that track within-person fluctuations, or employing purpose-built state measures that more cleanly isolate situational entitlement responses.

In addition, although Study 2 controlled for employee demography variables (i.e., gender, age, education, and organizational tenure), we did not measure other covariates that can also shape evaluations of coworkers, such as salary level and leader–member exchange (LMX). Future research could incorporate a broader set of individual and relational controls to further assess the robustness and boundary conditions of the present findings. In addition, processes beyond those tested here may matter. For example, high interdependence can raise questions about contribution attribution and credit allocation, which might elicit negative reactions even when knowledge seeking also occurs. Examining such parallel mechanisms would further clarify the interpersonal consequences of AI-augmented performance.

Third, the present research was conducted within a specific organizational and cultural context, limiting the generalizability of the findings. The psychological entitlement pathway documented here may operate differently in collectivist cultural contexts, where group-level harmony norms may suppress the open expression of entitlement cognitions even when such processes are internally activated, or in organizational cultures with strong AI usage norms that reframe AI-assisted performance as a collective rather than individual achievement. Future cross-cultural and cross-industry research is needed to establish the contextual boundary conditions of the proposed framework and to identify the cultural configurations under which the dual-path model holds most robustly.

Finally, the present study captures a snapshot of social comparison dynamics at a particular moment in organizations’ AI adoption trajectories. As AI tools continue to diffuse and coworker AI-augmented performance becomes increasingly commonplace, the salience and intensity of comparison processes triggered by such performance may evolve. Longitudinal research tracking teams over extended periods of AI adoption would shed light on whether the psychological entitlement pathway attenuates as AI-assisted performance becomes normative—effectively reducing the distinctiveness of any single coworker’s AI-augmented performance as a comparison stimulus—and whether the learning pathway becomes more institutionalized through team-level norms that explicitly encourage knowledge sharing around AI practices.

## Conclusion

Drawing upon social comparison theory, we found that coworker AI-augmented performance is a double-edged sword whose consequences are contingent upon task interdependence: it fosters interpersonal knowledge seeking and enhances work performance when interdependence is high, yet cultivates psychological entitlement and undermines performance when interdependence is low. By integrating these opposing pathways into a single theoretical framework, we hope the current study sparks future research on how the social diffusion of AI-enabled performance shapes individual and collective outcomes in organizations.

## Data Availability

The raw data supporting the conclusions of this article will be made available by the authors, without undue reservation.
